# Comparative transcriptomic analysis revealed novel potential therapeutic targets of traditional Chinese medicine (Pinggan-Qianyang decoction) on vascular remodeling in spontaneously hypertensive rats

**DOI:** 10.1186/s13020-021-00431-4

**Published:** 2021-02-10

**Authors:** Jiamei Yao, Cui Zhang, Yushu Yang, Xia Fang, Qiong Chen, Guangwei Zhong

**Affiliations:** 1 Department of International Medical Center, Xiangya Hospital, Central South University, 87 Xiangya Road, Kaifu District, Changsha, 410008 China; 2 National Clinical Research Center for Geriatric Disorders, Xiangya Hospital, Central South University, 87 Xiangya Road, Kaifu District, Changsha, 410008 China

**Keywords:** Traditional Chinese medicine, Comparative transcriptomic analysis, Vascular remodeling, Spontaneously hypertensive rats, AGE–RAGE signal pathway, Cell autophagy

## Abstract

**Background:**

Both experimental and clinical studies have revealed satisfactory effects with the traditional Chinese formula Pinggan Qianyang decoction (PGQYD) for improving vascular remodeling caused by essential hypertension. The present study explored various therapeutic targets of PGQYD using mRNA transcriptomics.

**Methods:**

In this study, rats were randomly divided into three groups: Wistar-Kyoto (WKY; normal control), spontaneously hypertensive (SHR), and PGQYD-treated rat groups. After 12 weeks of PGQYD treatment, behavioral tests were employed and the morphology of thoracic aortas were examined with hematoxylin–eosin (HE) and Masson staining and electron microscopy. The mRNA expression profiles were identified with RNA-Seq and quantitative real-time PCR to validate changes in gene expression observed with microarray analysis. The gene ontology and pathway enrichment analyses were carried out to predict gene function and gene co-expressions. Pathway networks were constructed to identify the hub biomarkers, which were further validated by western blotting and immunofluorescence analysis.

**Results:**

After PGQYD treatment, the behavioral tests and histological and morphological findings of vascular remodeling were obviously meliorated compared with the SHR group. In the rat thoracic aorta tissues, 626 mRNAs with an exact match were identified. A total of 129 of mRNAs (fold change > 1.3 and P-value < 0.05) were significantly changed in the SHR group compared to the WKY group. Among them, 16 mRNAs were markedly regulated by PGQYD treatment and validated with quantitative real-time PCR. Additionally, target prediction and bioinformatics analyses revealed that these mRNAs could play therapeutic roles through biological processes for regulating cell metabolic processes (such as glycation biology), biological adhesions, rhythmic processes, and cell autophagy. The cellular signaling pathways involved in autophagy may be AGE–RAGE/PI3K/Akt/mTOR signaling pathway.

**Conclusion:**

The present study provides novel insights for future investigations to explore the mechanisms by which PGQYD may effectively inhibit vascular remodeling by activating the AGE–RAGE/PI3K/Akt/mTOR signal pathway in cell autophagy biology.

## Background

Essential hypertension (EH) is a major public health problem both in middle-aged and elderly people. It is both a complex disease and an important risk factor for other cardiovascular outcomes, such as sudden death, stroke, myocardial infarction, heart failure, and renal diseases [1]. Unfortunately, the control of arterial hypertension is far from optimal and has improved only minimally over the last few decades [2]. Side effects of anti- hypertensive drugs, complaints due to their blood pressure lowering effect, and inadequate compliance are the key factors in the background of inadequate control of hypertension [3, 4]. Therefore, it is urgent to find new antihypertensive therapy in the future. The spontaneously hypertensive (SHR) rat model is a suitable model for studying the development and consequences of hypertension. The development of vascular remodeling is an early and important consequence of hypertension. Vascular remodeling is mainly characterized by vascular smooth muscle cell hypertrophy and increased production of the extracellular matrix [5]. Vascular remodeling is initially an adaptive process that evolves in response to long-term pressure overload, but later it can contribute to the development of hypertensive target organ damage [6].

The development of new effective therapies for EH is urgently required. Fortunately, traditional Chinese medicine (TCM), with more than a 2000-year history, includes some of the oldest herbal medicines in the world [7]. Based on a multi-component and multi-target approach, TCM has become an important source for new drug development to treat essential hypertension [8, 9]. The World Health Organization encourages the incorporation of herbal remedies into main stream medical systems [10]. Pinggan Qianyang decoction (PGQYD), a famous TCM formula, is by far the most frequently used for the treatment of EH (87.9%) [11]. Accumulating evidence has suggested that PGQYD improves target organ damage of EH through vascular protection, vascular anti-aging effects, and improvement in vascular remodeling [12]. Moreover, our previous study confirmed that PGQYD tends to improve vascular remodeling by enhancing HSP27 expression through the p38MAPK signaling pathway [13]. However, the multifaceted regulatory mechanisms of PGQYD acting on EH have not been fully elucidated due to the lack of appropriate methods.

**Fig. 1:**
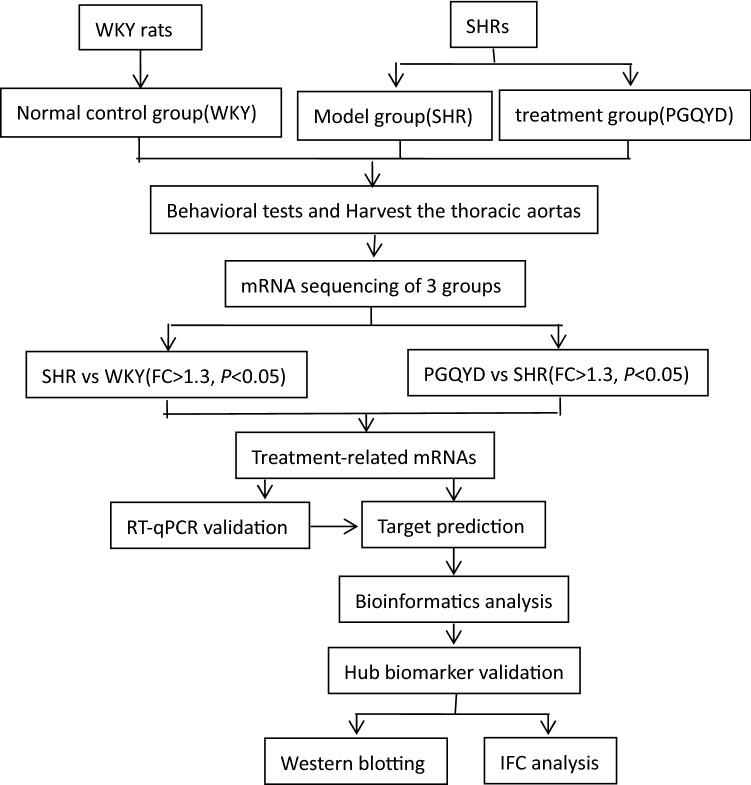
Experimental flow chart

Advanced omics technology is considered to be a holistic and efficient tool to study the use of TCM, and it can be used as a bridge between TCM and western medicine [14]. Transcriptomics is a powerful tool for detecting global alterations in RNA expression and, consequently, changes in the corresponding proteins [15, 16]. A transcriptomics study of Buyang-Huanwu Decoction (BYHWD) for treating intracerebral hemorrhage showed that three tRNAs (rno-tRFi-Ser-25a, rno-tRF5-Ala-16a, and rno-tRF5-Glu-29a) are specifically regulated by these drugs [17]. Another transcriptomics study of a TCM formula (Baoyuan decoction, BYD) for treating myocardial ischemia (MI) disclosed that the cardioprotection of BYD is mainly involved in the regulation of energy homeostasis, oxidative stress, apoptosis, inflammation, cardiac contractile dysfunction, and extracellular matrix remodeling [18]. Therefore, it is rational to believe that transcriptomics analysis of mRNAs may help shed light on multifaceted mechanisms of TCM formulas by identifying exact therapeutic targets and their interactions [19]. For that purpose, we aimed to utilize RNAsequencing technologies to evaluate the mRNA expression levels in Wistar-Kyoto (WKY; normal control) rats and the spontaneously hypertensive (SHR) and PGQYD-treated rats groups. Then hypertension-induced changed mRNAs (SHR vs WKY) and PGQYD-regulated altered mRNAs (PGQYD vs SHR) were obtained, and the key genes and pathways were identified with bioinformatics. Finally, the hub biomarkers were further validated by investigating protein expression levels. We evaluated their biological functions to reveal the therapeutic mechanisms of PGQYD (Fig. 1). These new findings may provide a novel perspective to illustrate the molecular mechanisms of PGQYD treatment for vascular remodeling after hypertension.

## Materials and methods

### Preparation of PGQYD

The ingredients of PGQYD included five medicinal herbs: Tian Ma (*Gastrodiae Rhizoma*), Gou Teng (*Uncaria Rhynchophylla*), Shi Jue Ming (*Haliotis Discus Hannai)*, Mao Li (*Ostrea Gigas Thunberg*), and Niu Xi (*Cyathulae Radix*) at a ratio of 10:20:30:30:20, respectively (dry weight). All herbs were obtained from Xiangya Hospital, Central South University (CSU; Changsha, China). Furthermore, each herb was authenticated by the herbal medicinal botanist, Professor Shao Liu, in the Department of Pharmacy at Xiangya Hospital, CSU. The decoction was prepared and subjected to quality control as previously described [20]. Finally, the powder was dissolved in distilled water at a concentration of 0.2 g/ml for intragastric administration.

### Animals and experimental groups

Twenty healthy male spontaneous hypertensive rats [14 weeks old, SCXK (Hu) 2018-009] and 10 healthy male WKY rats [14 weeks old, SCXK (Hu) 2018-009] weighing 220 − 240 g were purchased from Shanghai Slake Experimental Animal Co., Ltd. All the animals were reared under identical conditions (room temperature at 25 °C and 12-h light–dark cycle) and had free access to standard rodent water and food. All animal protocols were approved by the Committee on the Use and Care of Animals of CSU and conformed to the Guidelines for the Care and Use of Laboratory Animals. After 1 week of adaptive feeding, the SHR rats with systolic blood pressure (SBP) more than 165 mmHg and the WKY rats with SBP less than 120 mmHg were used in this investigation. The first group was an age-matched normotensive control group (WKY, n = 10). The SHR rats were randomly divided into two groups. One group received no treatment (SHR, n = 10), whereas the other group (PGQYD, n = 10) received 0.7 g/kg/day as the dosage of PGQYD for 4 weeks.

### Assessment of behaviors and measurement of blood pressure

The degree of irritability was observed and graded [21]: 1 point: screamed and jumped when holding the neck; 2 points: bit while holding the neck; 3 points: when the tail was raised, the rat screamed, jumped, and even bit the rats in the same cage, frequently fought, and bit the iron cage. If the above situation was not obvious, it was scored as level 0. The rotation tolerance time was measured. The rats were placed on a home-made rotating platform with a rotating speed of 45 r·min-1. If the rats did not fall after rotating for 2 min, the experiment was stopped. SBP was determined in conscious rats using the tail-cuff method [22]. The rat tails were occluded with the appropriate size tube-shaped tail cuff connected to the tail cuff device. The SBP and HR of the rats were measured via an XH200 thermostatic non-invasive blood pressure meter and an MD3000 biological signal collection and processing system (Huaibei Zhenghua Biological Instrument Equipment Co., Ltd., Anhui, China). Each result was averaged from three repeats at the beginning of the study, and at 1 week, 2 weeks, 3 weeks, and 4 weeks subsequently until euthanasia. On the 28th day, rats were deeply anesthetized by intraperitoneal injection of pentobarbital and perfused with ice-cold saline. Then, thoracic aortic tissues were harvested for subsequent analysis.

### Histological and morphological assay

Rats were anesthetized with an intraperitoneal injection of 5 mL/kg 20% urethane at the end of each week of whole-day drug administration. The thoracic aorta below the aortic arch of each rat was stripped and clipped. A portion was fixed in 8% neutral formaldehyde, embedded in paraffin, sectioned at 5 µm, and stained with hematoxylin–eosin (HE) and Masson, and then observed with electron microscopy [23]. Light microscopy was used to image each cross-sectional slice, of which there were five per rat. Each vascular ring in the perpendicular position and the vessel media wall was observed. The images were observed under a Leica imaging system (Leica Microsystems GmbH, Wetzlar, Germany). The media thickness (MT) and inner diameter (LD) were measured, and the ratio of media thickness to inner diameter (MT/LD) was calculated. Other parts of the thoracic aorta were removed from the adventitia and were promptly refrigerated at − 80 °C for processing in an mRNA assay.

### Transcript profile analysis

The total RNA of thoracic aorta tissues from nine rats (WKY group, n = 3, SHR group, n = 3, and PGQYD group, n = 3) were extracted using TRIzol (Invitrogen, Carlsbad, CA, USA) reagent for RNA sequencing and were purified according to the manufacturer’s instructions. Total RNA was extracted from the right globus pallidus and purified with an RNeasy Mini Kit (Qiagen, Redwood City, CA, USA). Arraystar Rat mRNA microarrays (v2.0, containing 24,626 coding transcripts) were used to detect the expression of mRNAs in a total of nine rats (three groups with three replicates). Tissue preparation and microarray hybridization were performed with an Agilent Gene Expression Hybridization Kit (Agilent Technology, CA, USA) [24]. After washing, the arrays were scanned with an Agilent Microarray Scanner and finally analyzed with Agilent Feature Extraction software (version 10.5.1.1). Differentially expressed transcripts (thresholds of ≥ 1.3-fold and *P* values of < 0.05) were identified by comparing the SHR model and normal control groups (SHR vs WKY) and the SHR + PGQYD-treated and SHR groups (PGQYD vs SHR). Then, we determined the intersection between the upregulated transcripts in the SHR vs WKY groups and the downregulated transcripts in the PGQYD vs SHR groups as well as the intersection between the downregulated transcripts in the SHR vs WKY groups and the upregulated transcripts in the PGQYD vs SHR groups to identify the mechanism by which PGQYD reverses pathophysiological changes in SHR.

### Analysis of differentially expressed genes (DEGs)

The expression level for each gene was determined by the number of reads uniquely mapped to the specific gene and the total number of uniquely mapped reads in the sample. The gene expression level was calculated with the reads per kb per million (RPKM) method [25]. The formula to calculate FPKM was as follows: FPKM = (number of mapping fragments) × 10^3^ × 10^6^/[(length of transcript) × (number of total fragments)]. Then, the NOI seq method was applied to screen DEGs between two groups, with the threshold of significance as fold change of RPKM ≥ 3 and probability ≥ 0.8 [26].

### Pathway enrichment analysis and GO analysis

GO (http://www.geneontology.org) analysis was performed to determine biological roles based on the molecular functions, biological processes, and cellular components of the aberrantly expressed mRNAs; *P* < 0.05 and false discovery rate (FDR) < 0.05 were used as thresholds to define markedly enriched GO terms/pathways [27]. Pathway analysis (based on KEGG, http://www.genome.jp/kegg/) was performed to explore the pathways significantly enriched in DEGs [28].

### Real-time quantitative PCR validation

The procedure of real-time quantitative PCR was based on a previous study [29]. Total RNA was extracted from tissues in each group and 5 µg of total RNA was reversely transcribed to cDNA (Tiangen Biotech, Shanghai, China) based on the manufacturer’s manuals. RT-qPCR was performed in a reaction system (25 µL) that contained SYBR Green/Fluorescein qPCR Master Mix, forward primers, reverse primers, and cDNA. The ABI7500 realtime PCR system (provided by Applied Biosystems, CA, USA) was employed for PCR. Alterations in mRNA expression in thoracic aortas tissues were assessed with the 2^−△△Ct^ method normalized with endogenous control GAPDH. The PCR primers that were used are listed in Additional file 1: Table S1.

### Immunofluorescence (IFC) analysis

The staining procedure for IFC analysis was based on a previous study [30]. The 5 µm acetone-fixed thoracic aorta frozen sections were incubated for 1 h in 10% bovine serum to block nonspecific protein–protein interactions. They were cultured in PBS (overnight at 4 °C) and incubated with primary antibodies (rabbit anti-rat RAGE antibodies, 1:200, Abcam, Cambridge, MA, UK; rabbit anti-rat EGR-1 antibodies, 1:200, Abcam, Cambridge, UK). After washing three times for 10 min, the sections were incubated in PBS for 1 h at room temperature with secondary antibodies [goat anti-rabbit Alexa Fluor 594 (1:200)]. The nuclei were counterstained with 4-0-6-diamidino-2-phenylindole (DAPI; Invitrogen, Carlsbad, CA, USA). The immunofluorescence staining was analyzed using a laser-scanning confocal microscope (SLM 510, Carl Zeiss Meditec, Inc., Jena, Germany).

### Western blotting analysis

From the frozen thoracic aorta tissues, proteins were extracted and the concentrations were measured with a bicinchonininc acid (BCA) protein assay. Samples were subjected to incubation with primary antibodies antiCaSR (provided by Abcam; 1:800), renin (provided by Bioss, Beijing, China; 1:250), AT2R (provided by Abcam; 1:800), angiotensin II type 1 receptor (AT1R) (provided by Abcam; 1:800), MasR (Abcam; 1:800), B cell leukemia-2 (Bcl-2) (provided by Cell Signaling Technology, Danvers, USA; 1:1000), Bcl-2- associated X protein (Bax; provided by Cell Signaling Technology; 1:800), and caspase-3 (provided by Abcam; 1:500), and normalized to GAPDH (provided by Sugisuke Bridge, China; 1:1000). Bio-Rad Quantity One software (provided by Bio-Rad) was used to quantify the intensity of the protein bands. The primary antibodies used are listed in Additional file 1: Table S2.

### Statistics analysis

Statistical analyses were carried out with SPSS 19.0 (SPSS Inc., Chicago, IL, USA). Assays were conducted at least three times unless otherwise stated. All values were expressed as either the mean ± standard deviation (SD) or the mean ± the standard error (SE), except for the neurobehavioral scores. The comparison of data between groups was assessed using a one-way analysis of variance (ANOVA) followed by Tukey’s multiple-comparison test. Neurobehavioral scores were expressed as the median (interquartile range, IQR) and were analyzed using the Mann–Whitney U test. *P* < 0.05 was considered as statistical significance.

## Results

### Effects of PGQYD on behaviors and blood pressure in SHRs

**Fig. 2:**
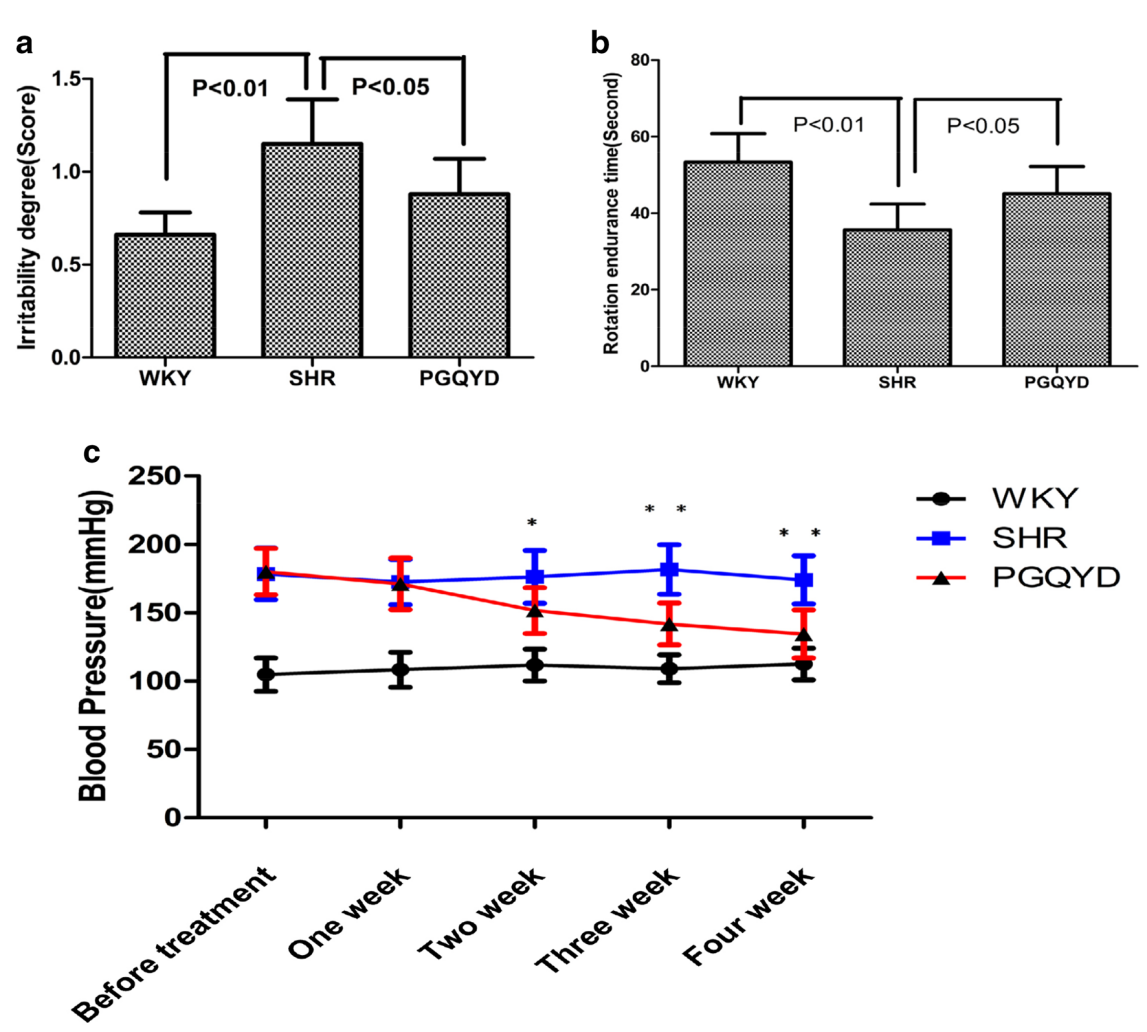
Effect of PGQYD on behavior and blood pressure in SHR rats. **a** Shows the comparison of irritability of the rats; **b** shows the comparison of rotation tolerance times in the rats; **c** shows the comparison of systolic blood pressure in the rats. Data are presented as the mean ± SEM (n = 10 each group), *P < 0.05 and **P < 0.01 compared with the PGQYD group

As shown in Fig. 2a, b, compared with the WKY group, the degree of irritability in the SHR group was significantly increased, while the rotation tolerance time was dramatically decreased (*P* < 0.01). Compared with the SHR group, the degree of irritability in the PGQYD group was significantly decreased (*P* < 0.05), and the rotation tolerance time was obviously prolonged (*P* < 0.05). The results showed that PGQYD could improve the behavior of SHR rats. As shown in Fig. 2c, before treatment, the blood pressure of the two groups of SHRs was significantly increased compared with that of the WKY group (*P* < 0.01). Compared with the SHR group, systolic blood pressure was reduced in the PGQYD group after 2 weeks (*P* < 0.05), and the efficacy was stable with the extension of the course of treatment (5th week). It was suggested that PGQYD could reduce the systolic blood pressure of SHR rats.

### Effect of PGQYD on vascular remodeling in the morphology of SHRs

**Fig. 3:**
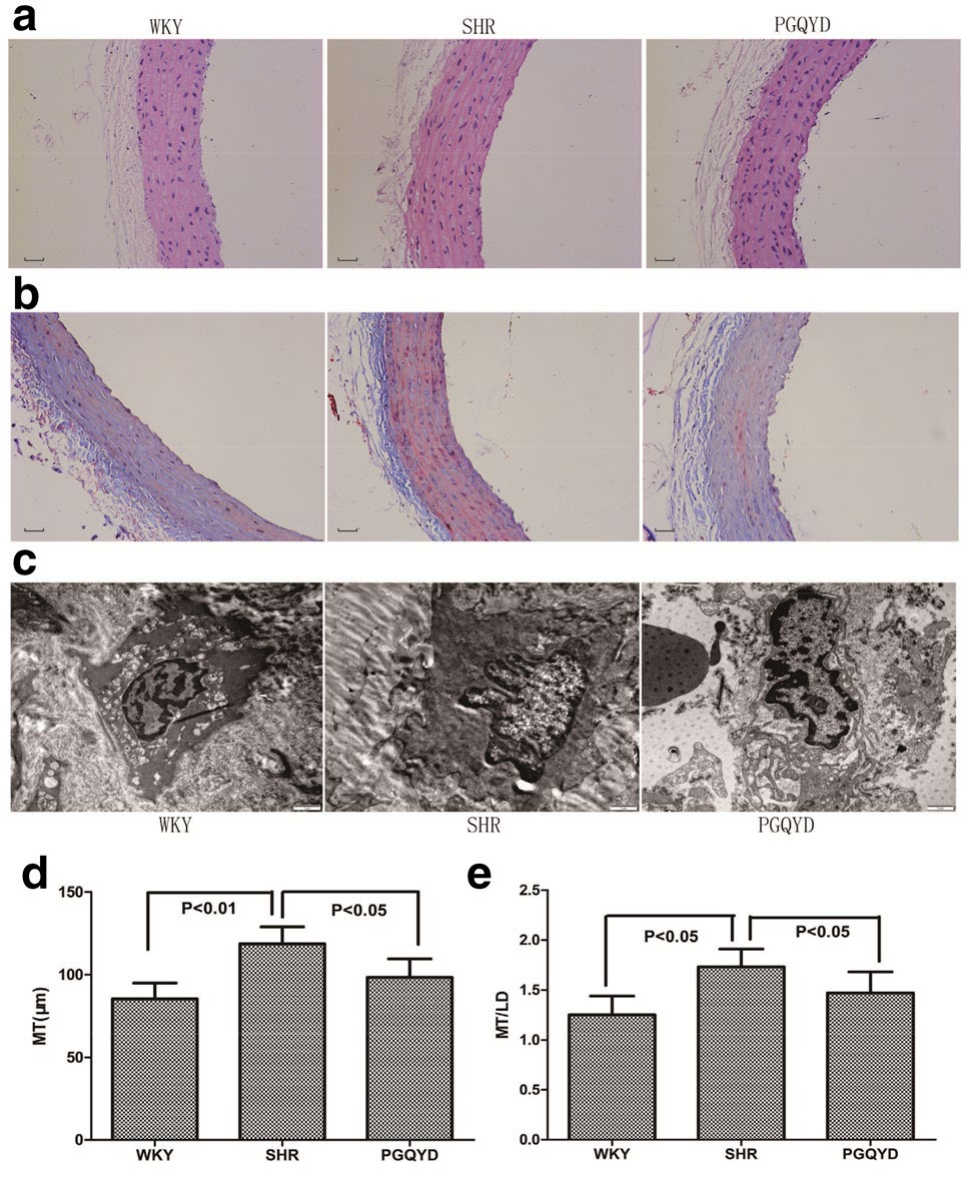
Effect of PGQYD on vascular remodeling in the morphology of SHR rats. **a** HE staining of a rat thoracic aorta (scale bar: 20 μm); **b** masson staining of a rat thoracic aorta (scale bar: 20 μm); **c** electron microscope observation of rat thoracic aortic vascular tissue (scale bar: 1 μm); **d** comparison of the media thickness (MT) of the thoracic aorta in rats; **e** the media thickness to the luminal internal diameter ratio (MT/LD) in the thoracic aortic vessel wall

Masson and HE staining showed that the aortic tunica media of the SHR group was thicker than that of WKY group, and the aortic tunica media of PGQYD-treated rats was thinner than that of the SHR rats in the SHR group (Fig. 3a, b). As shown in Fig. 3d, e, both MT and MT/LD were higher in the SHR group than in the WKY group (MT: 118.6 ± 10.3 µm vs 85.3 ± 9.6 µm, respectively, *P* = 0.009; MT/LD: 1.83 ± 0.18 vs 1.25 ± 0.19, respectively, *P* = 0.02). However, both MT and MT/LD were significantly lower in the PGQYD group than in the SHR group (MT: 98.3 ± 11.2 µm vs 118.6 ± 10.3 µm, respectively, *P* = 0.02; MT/LD: 1.47 ± 0.21 vs 1.83 ± 0.18, respectively, *P* = 0.04).

Comparison of rat aortic electron microscopy results in each group are shown in Fig. 3c. In the WKY group, organelles in the cytoplasm of vascular smooth muscle cells were not well developed, mitochondria were occasionally seen, and the endoplasmic reticulum was visible. In the SHR group, organelles in the cytoplasm of vascular smooth muscle cells were more developed, more mitochondria were seen, and the endoplasmic reticulum was visible. In the PGQYD group, organelles in the plasma of vascular smooth muscle cells were more developed, and the mitochondria and endoplasmic reticulum were visible. It was suggested that PGQYD could improve morphological changes in the aortic vascular tissue and reverse vascular remodeling in SHR rats.

### Classification of the transcriptomic phenotypes and identification of the altered transcripts with PGQYD treatment

**Fig. 4:**
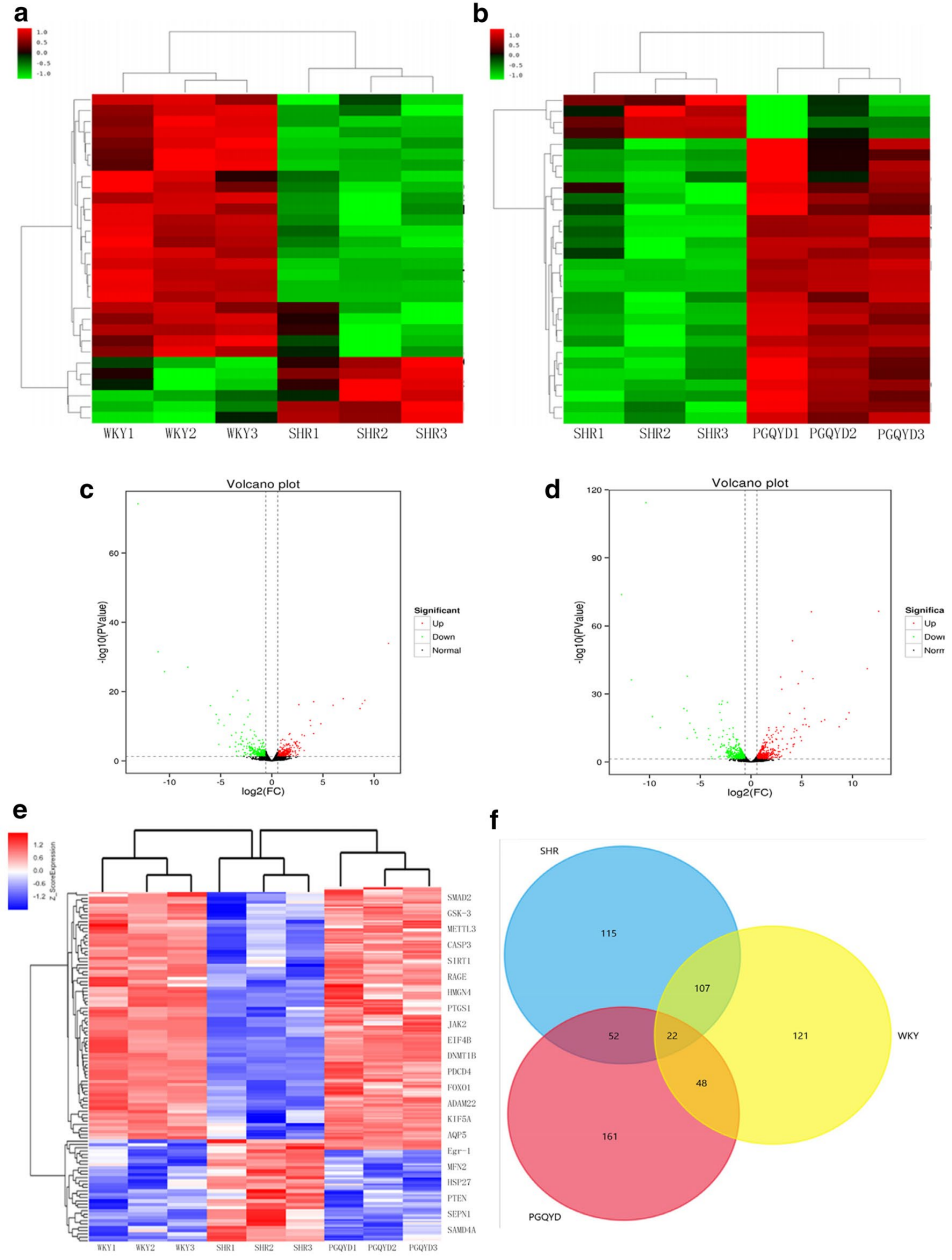
The differentially expressed genes among the groups. Hierarchical clustering between the WKY and SHR groups (**a**) and SHR and PGQYD groups (**b**). Green color represents downregulated genes, red color represents upregulated genes (P < 0.05). The Venn diagram of upregulated and downregulated genes between SHR/WKY (**c**) and PGQYD/SHR (**d**). The significantly changed mRNAs are shown in the heat map for the three groups (**e**); a Venn plot shows the total number of identified mRNAs in the thoracic aorta tissues of the WKY, SHR, and PGQYD groups (**f**)

A non-target global gene expression analysis was employed to explore how vascular remodeling altered aortic tissue mRNA expression, as well as evaluate the intervention with the PGQYD treatment on SHR rats. According to the cut-off criteria of |log2-fold change (FC)|> 1 and FDR < 0.05, a total of 465 DEGs were detected in the SHR group when compared to the WKY group (Fig. 4a, c), and a total of 511 DEGs were detected in the PGQYD group when compared to the SHR group (Fig. 4b, d). There were 129 and 70 DEGs (upregulated and downregulated, respectively) in the SHR group (relative to the WKY group) and PGQYD-treat (relative to the SHR group) group (Fig. 4e, f, respectively). Interestingly, PGQYD treatment could reverse these changes in the SHR group and created expression profiles close to WKY levels (Fig. 4f). Additionally, we identified 22 significantly dysregulated mRNAs in the SHR group: 16 were upregulated, while six were downregulated (SHR vs WKY). After PGQYD treatment, 22 mRNAs were obviously changed: six were upregulated, while 16 were downregulated (PGQYD vs SHR).

**Fig. 5:**
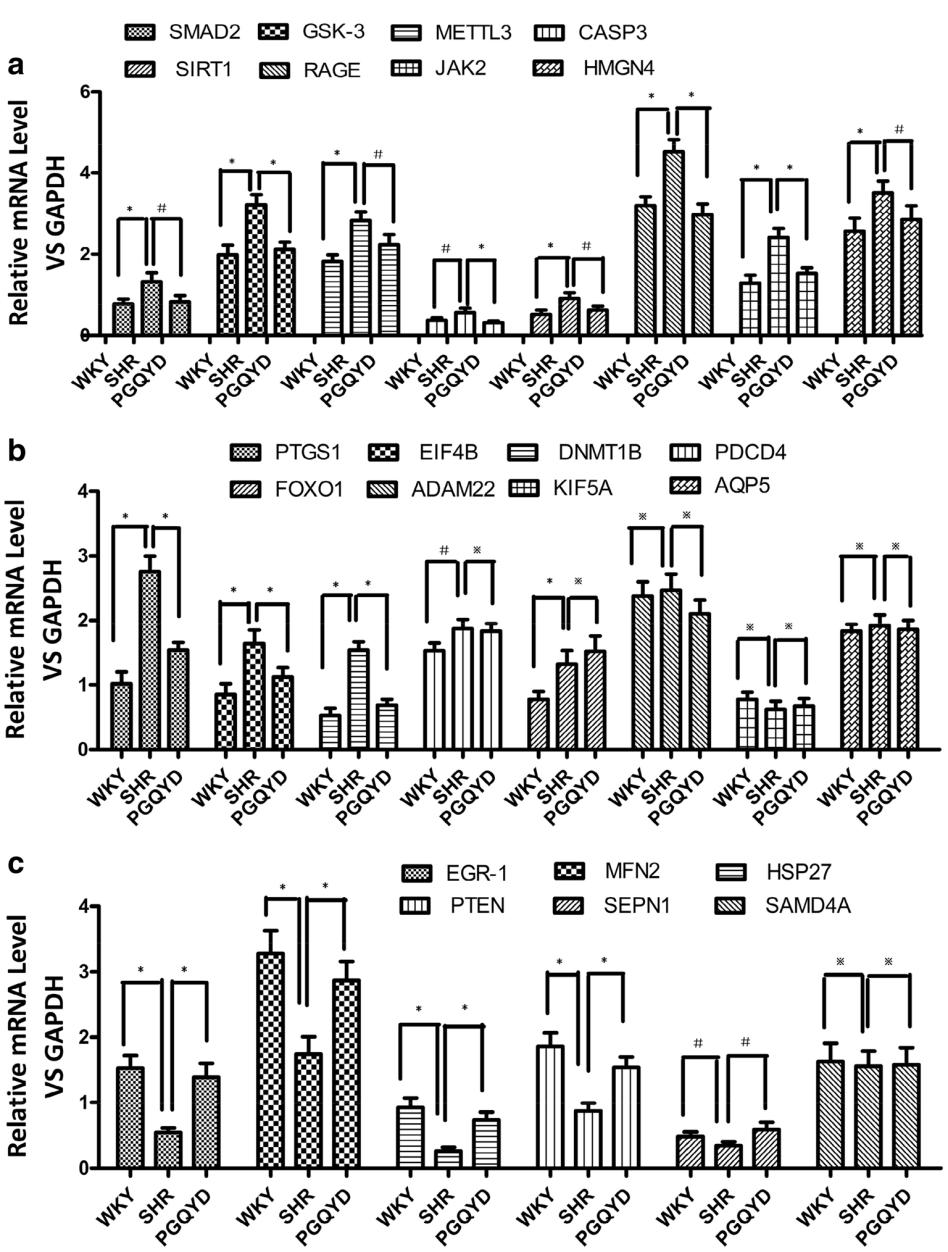
qRT-PCR confirmed the RNA-Seq data of the 22 RNAs using divergent primers. **a** Eight upregulated mRNAs. **b** Eight upregulated mRNAs; five upregulated mRNAs were not significantly different according to qRT-PCR methods. **c** Six downregulated mRNAs. Values are the mean ± SD (n = 5 per group). qRT-PCR analysis was conducted in triplicate. *P < 0.01, ^#^*P* < 0.05, ^※^*P* > 0.05 (Student’s t-test)

All expression levels of 22 candidate transcripts (Table 1) were validated at the transcript level by qRT-PCR (Fig. 5). The qRT-PCR results indicated statistically that the SHR group had increases in 11 gene expression levels (*SMAD2, GSK-3, METTL3, CASP3, SIRT1, RAGE, HMGN4, PTGS1, JAK2, EIF4B,* and *DNMT1*; Figs. 5a, b) and decreases in five gene expression levels (*Egr-1, MFN2, HSP27, PTEN,* and *SEPN1*; Fig. 5c).

**Table 1:** Symbol description

Abbreviations	Full name
SHR	Spontaneous hypertensive rats
WKY	Wistar-Kyoto rats
NO	Nitric oxide
ET	Endothelin
RAAS	Renin angiotensin aldosterone system
TCM	Traditional Chinese medicine
TpxII	Thioredoxin peroxidaseII
HSP27	Heat shock protein 27
ANXA1	Annexin A1
KEGG	Kyoto Encyclopedia of Genes and Genomes
AGE–RAGE	Advanced glycosylation end products–Receptor for advanced glycation end products
SMAD2	Smad2
PPAR-γ	Peroxisome proliferator activated receptor gamma
AT1R	Angiotensin II Type 1 Receptor
*JAK2*	Jak2
*CASP3*	Caspase 3
EGR-1	Egr-1
PGQYD	Pinggan Qianyang decoction
mTOR	Mammalian target gene of rapamycin
PI3K–Akt	Phosphorylated phosphatidylinositol 3 kinase
MAPK	Mitogen activated protein kinase
VSMC	Vascular smooth muscle cell
PDGF-BB	Platelet derived growth factor BB
JNK	C-Jun N-terminal kinase
MEF2D	Myocyte enhancer factor 2D
GSK3β	Glycogen synthase kinase-3β
AMPK	Adenosine 5ʹ-monophosphate (AMP)-activated protein kinase
DEGs	Differentially Expressed Genes

### Enrichment analysis of GO and KEGG pathways

**Fig. 6:**
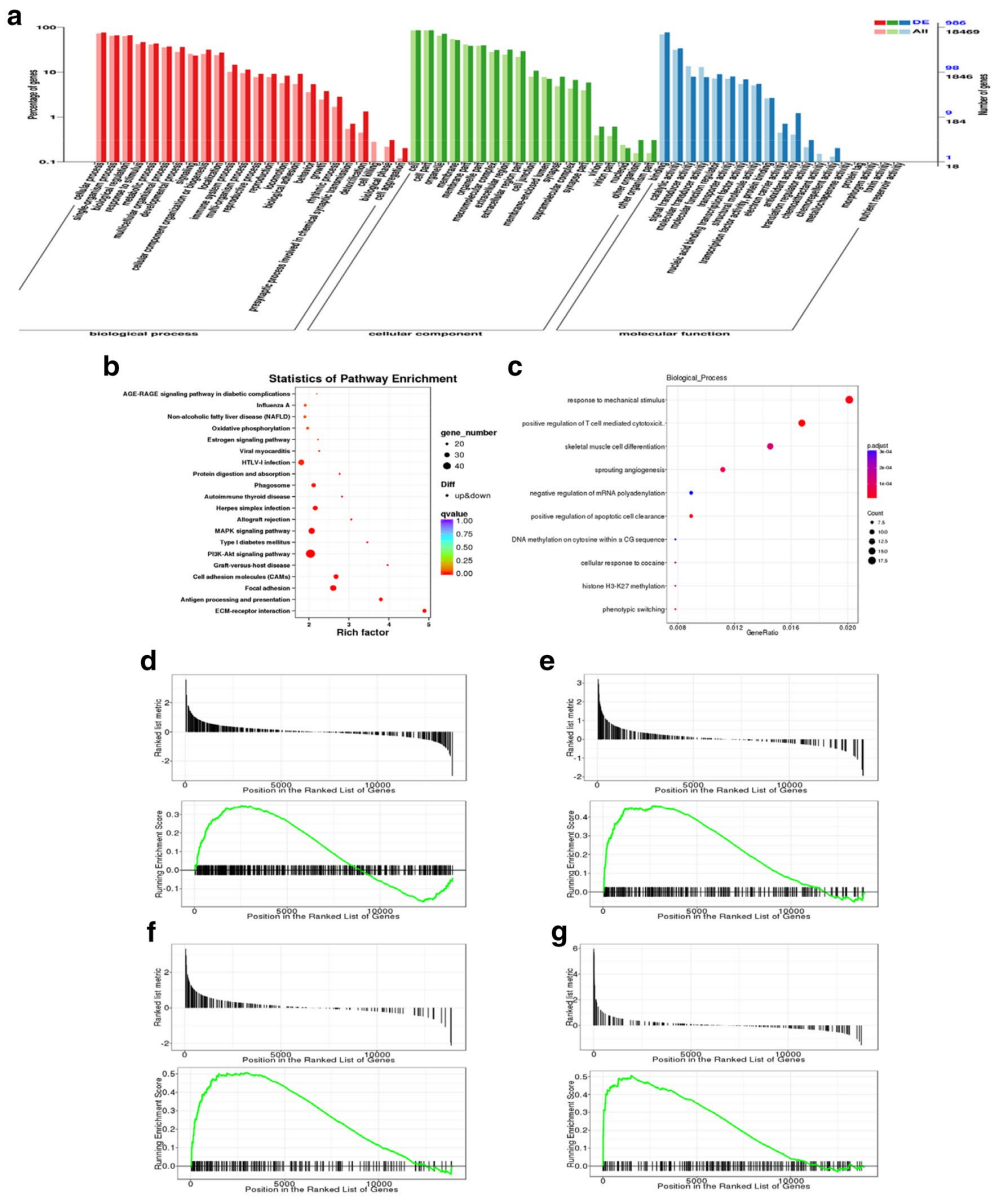
Analyzed pathways of transcriptomics data differently regulated in spontaneously hypertensive rats. **a** GO enrichment analysis of DEGs between the SHR model rats and WKY rats in biological processes (BP), cellular component (CC), and molecular function (MF). **b**–**c** GSEA analysis of DEGs between the SHR and WKY groups. **d**–**g** Gene set enrichment analysis (GSEA) with a high enrichment score for gene sets: **d** AGE–RAGE signaling pathway (**e**), oxidative phosphorylation (**f**), MAPK signaling pathway, and **g** PI3K–AKT signaling pathway

**Fig. 7:**
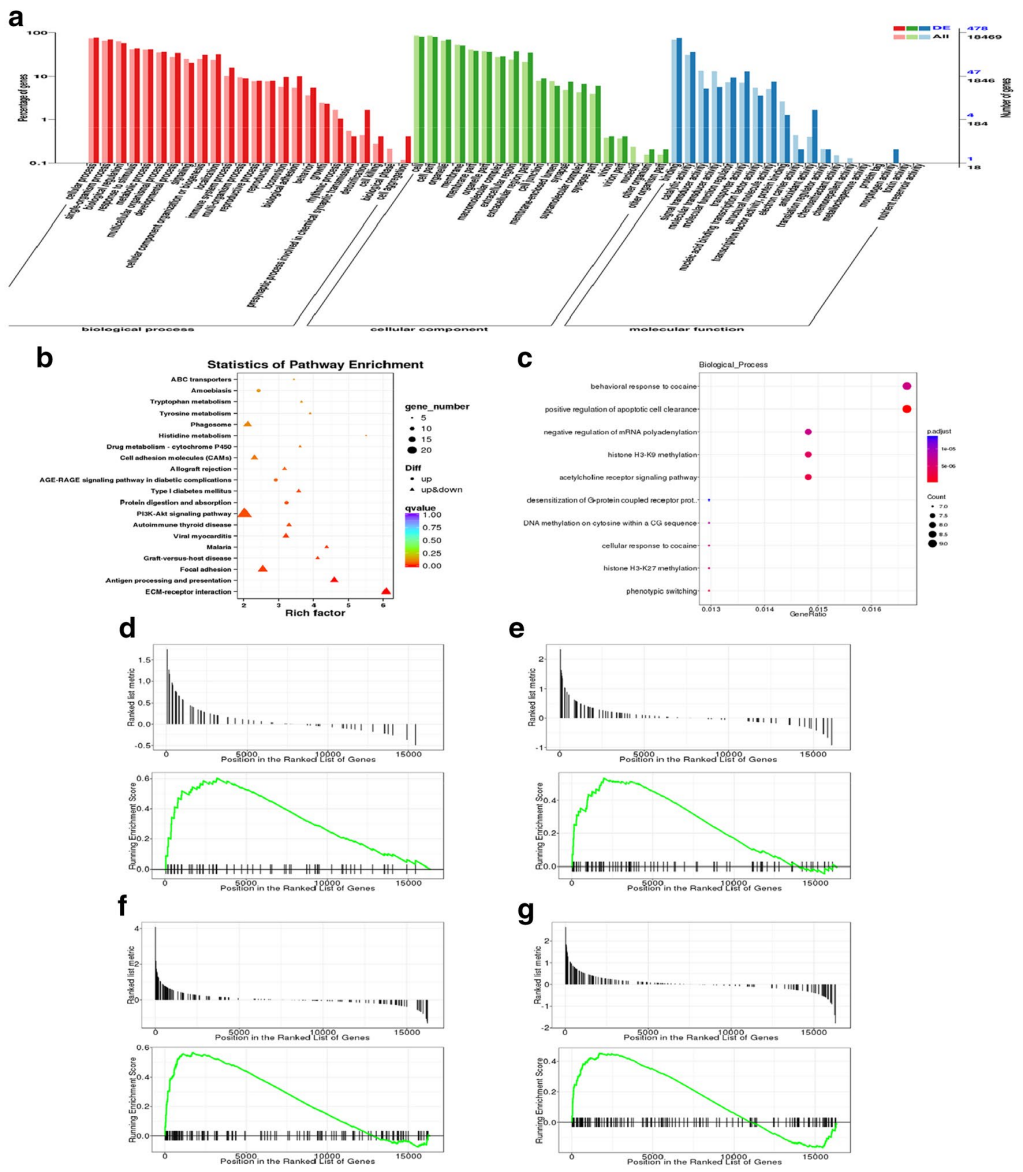
Analyzed pathways for transcriptomics data differently regulated in PGQYD-treated rats. **a** GO enrichment analysis of DEGs between the SHR model rats and the PGQYD group in biological process (BP), cellular component (CC), and molecular function M(F); **b**, **c** GSEA analysis of DEGs between the SHR and PGQYD groups; **d**–**g** Gene set enrichment analysis (GSEA) with a high enrichment score for gene sets; **d** AGE–RAGE signaling pathway; **e** phagosomes; **f** ECM-receptor interactions; and **g** PI3K–AKT signaling pathway

GO and KEGG assignments were used to classify the genes associated with the response of vascular remodeling to PGQYD treatment. GO classification analysis provided the percentage and distribution of top-level GO terms that were portrayed in three categories: (A) cellular component; (B) molecular function, and (C) biological process. As shown in Figs. 6a and 7a, we found that transcripts regulated in the thoracic aorta tissues of these model rats, as compared with the WKY group and PGQYD treatment group, could be mapped to biological processes (BP) for regulating cell metabolic processes, biological adhesions, rhythmic processes, cell autophagy, cell components (CC) for cell junctions, membrane parts and extracellular regions, molecular functions (MF) for receptor binding, signal transducer activity, antioxidant activity, transcription factor activity, and catalytic activity. For further characterization of the DEGs, we performed pathway enrichment analysis with GSEA. As shown in Fig. 6b–d, we found that transcripts regulated in the thoracic aorta tissues of these model rats, compared with the WKY group, could be mapped to signaling pathways, such as the AGE–RAGE signaling pathway, oxidative phosphorylation, MAPK signaling pathway, and PI3K–AKT signaling pathway. As shown in Fig. 7b–d, we found that transcripts regulated in the thoracic aorta tissues of these model rats, compared with the PGQYD treatment group, could be mapped to signaling pathways, such as the AGE–RAGE signaling pathway, phagosomes, ECM-receptor interactions, and the PI3K–AKT signaling pathway.

### Gene co-expression network analysis of the differences in the WKY, SHR, and PGQYD groups

**Fig. 8:**
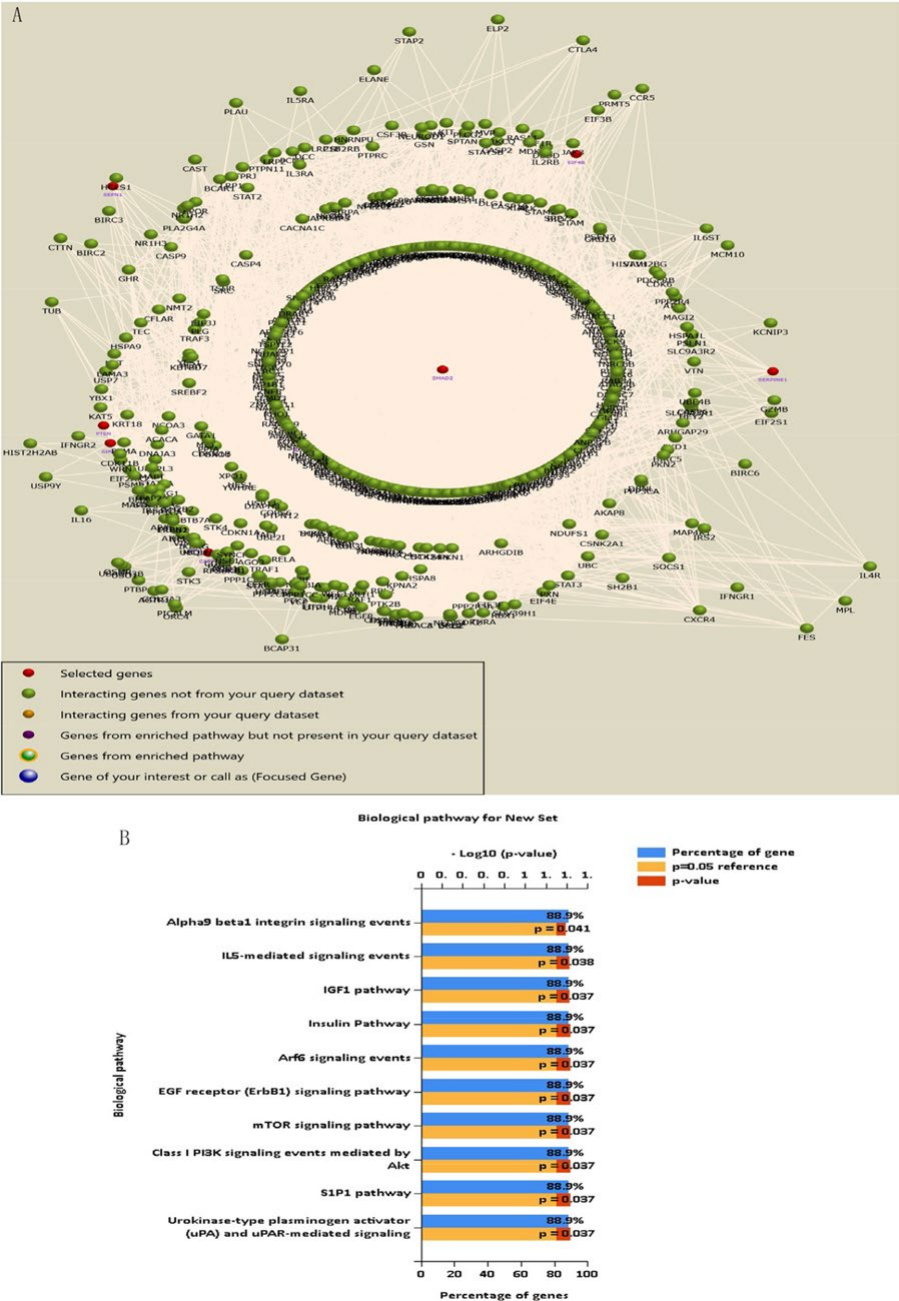
Integrated analysis of the mechanism of PGQYD-treated SHR rats from transcriptomics data. **a** Co-expression networks constructed by weighted correlation network analysis. Lines indicate interactions between pathways, and pathways indicated by the arrowhead were regulated by pathways of the arrow tail. Green nodes represent upregulated pathways and red nodes represent downregulated pathways. **b** Top 10 enriched signaling pathways are shown ranked by P-values and colored by the number of enriched genes (Additional file 1: Table S3)

According to transcriptome analysis of the three groups, 16 gene expressions were dysregulated (including 11 upregulated genes and six down regulated genes) and affirmed by qRT-PCR detection. These dysregulated genes were closely related to the vascular remodeling of hypertension treated by PGQYD. ESR1 was identified as the hub gene (Fig. 8a). Combined with the prediction of the PI3K–Akt signal pathway, AGE–RAGE signal pathway, and mTOR signal pathway were closely related to the vascular remodeling of hypertension treated by PGQYD (Fig. 8b, Additional file 1: Table S3). It was suggested that *GSK-3, eIF4B, PTEN, CCND1, SMAD2, JAK2, CASP3, EGR-1, RAGE,* and *FOXO1* were closely related to the above-mentioned signaling pathways.

### ***Validation of targeted genes enriched in the AGE***–***RAGE signaling pathway after PGQYD treatment***

**Fig. 9:**
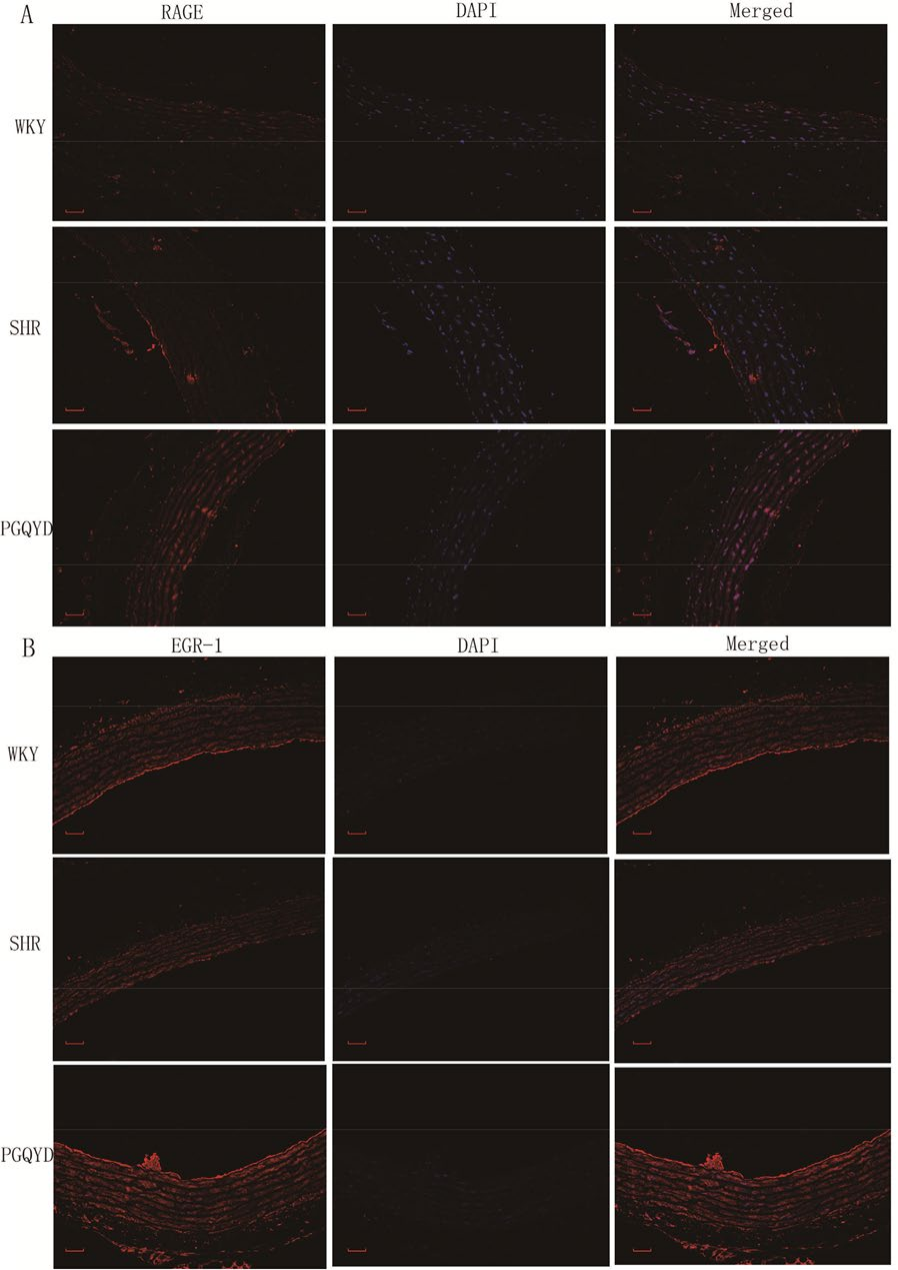
Immunofluorescence analysis was used to detect the levels of RAGE and EGR-1 in the thoracic aorta tissues. **a** Immunofluorescence staining of the antibodies to RAGE (red) in the thoracic aorta tissues of all groups (magnification, ×200, Scale bar: 20 μm). **b** Immunofluorescence staining of the antibodies to EGR-1 (red) in thoracic aorta tissues of all groups. DAPI (blue) was used to stain the nucleus (magnification, ×200, Scale bar: 20 μm)

**Fig. 10:**
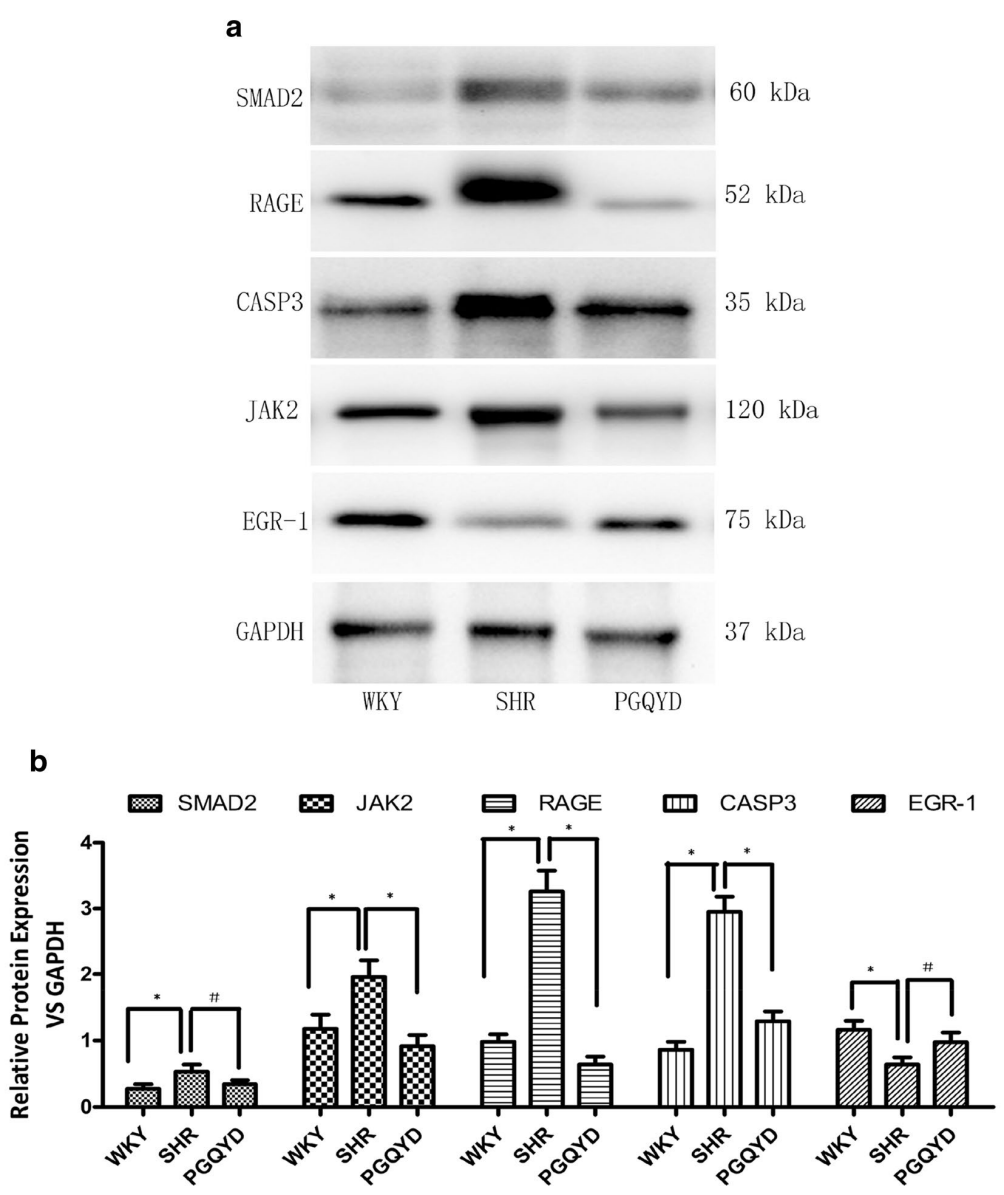
Validation of the hub genes in the AGE–RAGE signaling pathway. **a** Expression of *SMAD2, JAK2, RAGE, CASP3,* and *EGR-1* in thoracic aorta tissues were detected by western blotting. **b** The relative expression protein levels of *SMAD2, JAK2, RAGE, CASP3,* and *EGR-1*. *P < 0.01 compared with WKY group. *P < 0.01 compared with the SHR model group. ^#^P < 0.05 compared with the SHR model group

**Fig. 11:**
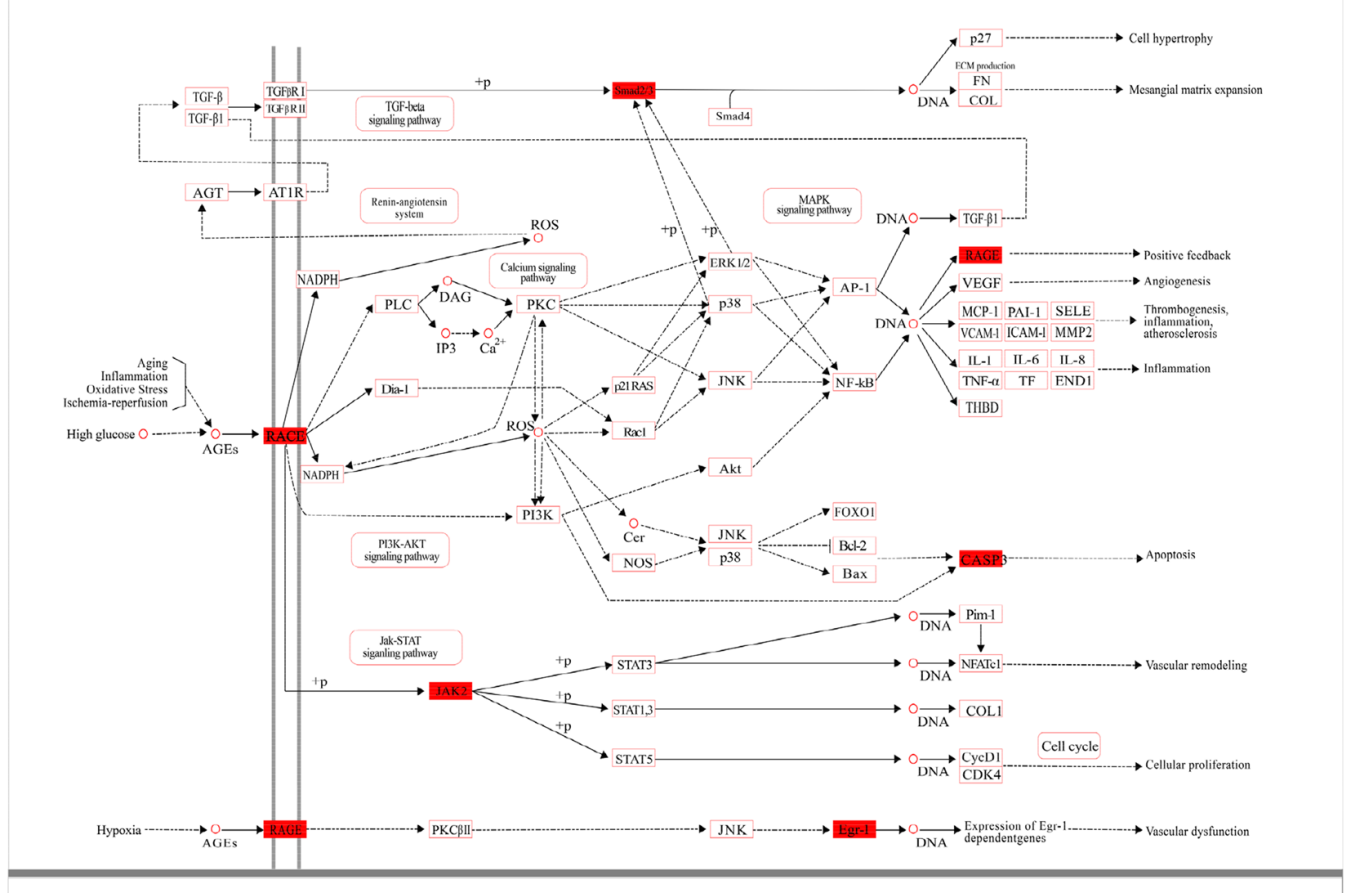
Five genes are indicated in red on the AGE–RAGE signaling pathway map after PGQYD treatment (red)

Six genes were enriched in the AGE–RAGE signaling pathway (*SMAD2, JAK2, RAGE, CASP3,* and *EGR-1*). These results indicated that the role of the AGE–RAGE signaling pathway was important in the SHR model rats after PGQYD treatment. Thus, the western blot method and immunofluorescence analysis were utilized to determine the expression levels of *SMAD2, JAK2, RAGE, CASP3,* and *EGR-1* in thoracic aorta tissues. The results showed that *SMAD2, CASP3, JAK2,* and *RAGE* expression levels were higher in the SHR group compared with the WKY group. However, after vascular remodeling injury, the expression levels of *EGR-1* were remarkably decreased (Fig. 9). To different extents, the PGQYD treatment improved the expression levels of these five proteins. Among them, *EGR-1* had remarkably increased protein expression levels (Fig. 10a). *SMAD2, CASP3, JAK2,* and *RAGE* had significantly decreased protein expression levels compared with the SHR group (Figs. 10b, c). The above results indicated that PGQYD effectively regulated the AGE–RAGE signaling pathway in vascular remodeling. Regulation of the AGE–RAGE signaling pathway was involved in the PGQYD pharmaceutic effect on hypertension, which agreed with the predicted mechanism mentioned previously (Fig. 11).

## Discussion

In this study, the SHR rats displayed remarkably enhanced vascular remodeling. Moreover, rat aortic electron microscopy images indicated that organelles in the cytoplasm of vascular smooth muscle cells were more developed, more mitochondria were seen, and the endoplasmic reticulum was visible compared to the WKY rats. Gastrodin can inhibit the proliferation of vascular smooth muscle cells, and delay aging [31]. In addition, Gastrodin can promote the endogenous vasodilator and inhibit the release of vasoconstrictor, thus showing a anti-hypertensive effect by increasing the synthesis of NO and decreasing the contents of ET and Ang-II [32]. The alkaloids in *U. rhynchophylla* can inhibit the proliferation and senescence of endothelial cells, thus protecting vascular endothelial cells and delaying vascular remodeling [33]. it can also increase the apoptosis rate of vascular smooth muscle cells, but inhibit its proliferation, showing an indirect inhibitory effect on vascular remodeling induced by Ang-II [34]. Studies had shown that ethanol extract of *Achyranthes bidentata* and *Cassia obtusifolia* can inhibit the activity of angiotensin-converting enzyme involved in RAAS system [35, 36]. It can affect the concentration of serum calcium ion, thus affecting the calcium channel, and has a strong and lasting anti-hypertensive effect, especially for those caused by long-term tension [37]. In the present study, our findings showed that PGQYD, a specific TCM for treating essential hypertension, could substantially improve blood pressure, behavioral, pathology (vascular remodeling) and endothelial dysfunction in essential hypertension, indicating there were unique advantages in the herb combinations and synergistic actions of multiple ingredients in the TCM.

Whether it is combined with western medicine to reduce blood pressure or alone, PGQYD had the advantages of multi-target, multi-channel, safe and effective. At present, the research on the mechanism of anti-hypertension has not been elucidated, mainly focusing on improving endothelial function, inhibiting the excessive activation of RAAS system, and improving the function of autonomic nervous system. We can further study its anti-hypertensive mechanism, anti-hypertensive pathway and the protective effect of other target organs by genomics and metabonomics. In the past, we had found that PGQYD could regulate the expression of some specific proteins(TpxII, HSP27 and ANXA1) and miRNAs(miR-20a, miR-145, miR-30, and miR-98) by used proteomics and miRNA gene chip technology [38, 39]. In this study, we first described the transcriptomic features of PGQYD-treated SHR rats, and KEGG analysis suggested that the regulated transcripts were attributed to multiple functions, such as cell metabolic processes, biological adhesions, rhythmic processes, and cell autophagy. Most genes involved in the three cellular functions were dysregulated. According to the pathway and gene co-expression network of PGQYD-specific genes, the AGE–RAGE signaling pathway, PI3K–Akt signal pathway, and SMAD2 were at the core of the networks.

Some evidence had supported the view that the glycation of proteins was one of the main factors contributing to vascular remodling and cardiac target organ damage in mild hypertensives [40, 41]. Treatment with sRAGE had alleviated vascular adverse remodeling in SHR, possibly via suppression of oxidative stress and inflammation, improvement in RAS balance, and activation of PPAR-γ pathway [42]. The present study had showed that the AGE–RAGE may have close relationship with the pathogenesis of Qi and Yin deficiency and blood stasis in diabetic nephropathy patients [43]. The natural product gastrodin had potent activities in lowering blood glucose, improving insulin resistance and ameliorating diabetic nephropathy by reducing the contents of advanced glycation end product (AGE) in renal tissues [44]. In this study, the results suggested that the process of cell metabolism in glycation may play an important role in the vascular remodeling with PGQYD treatment.

Blood pressure exhibits a robust circadian rhythm in health. This balanced rhythm in hypertension is perturbed via elevations in night-time blood pressure, inflicting silent damage to the vasculature and body organs. These data suggest that circadian clock genes(Per2, bmal1) may act to inhibit or control renin/angiotensin signaling [45]. Chinese herbal medicine for PGQYD may lower the blood pressure smoothly and recover the circadian rhythm of blood pressure in essential hypertension patients related to the regulation of vascular endothelium function [12]. Tianma Gouteng Yin can regulate the circadian rhythm of clock gene in vascular smooth muscle cells induced by AngII through AT1R signaling pathway [46]. Isorhynchophylline was the main alkaloids in *U. rhynchophylla* had certain effect on regulating the disordered circadian rhythm of clock genes in vascular smooth muscle cells (A7r5 cell line) induced by Ang-II through AT1R-Src signaling pathway [47]. In this study, the results suggested that the process of rhythmic processes may play an important role in the vascular remodeling with PGQYD treatment.

In our study, these findings showed that the expressions of *RAGE*, *SMAD2*, *JAK2*, and *CASP3* were upregulated and the expression of EGR-1 was downregulated in the SHR model rats. Among them, five mRNAs were markedly regulated with PGQYD treatment. The results indicated that the mechanism for PGQYD reversing vascular remodeling may be involved in the process of glycation regulation, which was carried out by *RAGE*, *SMAD2*, *JAK2*, *CASP3*, and *EGR-1*. Through bioinformatics analysis, we found that five genes are involved in regulation through the AGE–RAGE signaling pathway, PI3K–Akt signaling pathway, and mTOR signaling pathway. The study showed that paeoniflorin inhibited autophagy at least partly by inhibiting RAGE and upregulating the level of p-mTOR to act against AGE-induced mesangial cell dysfunction.

Autophagy plays an important role in cellular waste removal and structural reconstruction [48]. Tanshinone IIA can inhibit Angiotensin II-induced proliferation and autophagy of vascular smooth muscle cells via regulating the MAPK signaling pathway [49]. The total alkaloids of *U. rhynchophylla* can reduce the apoptosis of endothelial cells by enhancing autophagy in SHR [50]. Gastrodin suppresses apoptosis possibly mediated by inhibiting autophagy in serum deprivation-stimulated H9C2 cardiomyocytes [51].The mTOR signaling pathway is involved in vascular remodeling against stress, such as pressure overload in VSMCs. Jin X et al. have shown that activation of the PI3K/Akt signaling pathway can reduce levels of reactive oxygen species and lipid deposition, thereby inhibiting endothelial dysfunction and reversing the progression of vascular remodeling [52]. Nε-Carboxymethyl-Lysine (the key active component of AGEs) significantly decreased the phosphorylation of PI3K/AKT signaling and restoration of PI3K/AKT signaling in cell apoptosis. The results showed RAGE induced foam cell apoptosis in diabetic atherosclerosis by inhibiting the PI3K/AKT pathway [53]. Rhynchophylline and Isorhynchophylline may promote the apoptosis and inhibit rhe proliferation and migration of VSMC by inducing Ang-II [54]. Gastrodin inhibits PDGF-BB induced VSMC migration, its mechanisms may be associated with the inhibition of the JNK signaling pathway activation [55]. The rhynchophylline can inhibite MPP^+^-triggered neurotoxicity by stimulating MEF2D via activating PI3-K/Akt/GSK3β signaling pathway [56].

The PI3K/Akt/mTOR signaling pathway is thought to be a crucial regulator of autophagy and apoptosis [57]. It has been confirmed that the mechanisms of apoptosis and autophagy in vascular smooth muscle cells were involved in vascular remodeling of hypertension [58]. It has also been confirmed that activation of the PI3K/Akt/mTOR pathway was detected in the proliferation, migration, and autophagy of VSMCs, which enhanced phenotype transformation [59]. Tianma Gouteng Decoction can significantly regulate the autophagy-related pathway Ca2 + /AMPK/mTOR of vascular endothelial cells in spontaneous hypertensive rats, which is probably one of the important mechanisms of its antihypertensive effect [60]. In our study, the results indicated that the PI3K/Akt/mTOR signal pathway was involved in the process of vascular remodeling, and this regulated the mechanism of PGQYD in reversing vascular remodeling. These findings suggested that the mechanism of PGQYD in reversing vascular remodeling was involved in the AGE–RAGE/PI3K/Akt/mTOR signaling pathway, which regulated the target gene expression and regulated the process of cell autophagy biology.

## Conclusions

In conclusion, we performed a transcriptomics analysis of gene expression changes in SHR rats under treatment with PGQYD using a RNA-seq technique. Overall, a total of 465 predicted protein-encoding genes were identified. Further gene expression analysis revealed a total of 16 DEGs, including 11 upregulated genes and five downregulated genes. Intensive bioinformatics analysis identified cell regulation, cell metabolic processes, biological adhesions, rhythmic processes, and cell autophagy as significant biological processes. The AGE–RAGE signaling pathway, PI3K–Akt signal pathway, and mTOR signal pathway were mainly involved. Overall, the results from this study might provide insights into the understanding of the mechanisms for the response to PGQYD. Furthermore, this work demonstrated the potential utility of the RNA-seq technique in ant- hypertension studies.

## Supplementary Information

**Additional file 1: Table S1.** Sequences of the Primers and Product size in this Study. **Table S2.** Primary antibody information. **Table S3.** Pathways enrichment analysis of differently expressed genes in SHR of PGQYD-treated rats.

## Data Availability

The datasets used during the current study are available from the corresponding author on reasonable request.
