# Supplementary material for: Comparative transcriptomic analysis revealed novel potential therapeutic targets of traditional Chinese medicine (Pinggan-Qianyang decoction) on vascular remodeling in spontaneously hypertensive rats
Source: Chin Med. 2021 Feb 10;16:21. doi: 10.1186/s13020-021-00431-4 (PMC7877093; doi:10.1186/s13020-021-00431-4)
Supplement: Supplementary file 1 — Additional file 1: Table S1. Sequences of the Primers and Product size in this Study. Table S2. Primary antibody information. Table S3. Pathways enrichment analysis of differently expressed genes in SHR of PGQYD-treated rats. [file 13020_2021_431_MOESM1_ESM.doc]

**Table S1 Sequences of the Primers and Product size in this Study**

| **Gene:NCBI Reference Sequence** | **Sequences** | **Product size(bp)** |
| --- | --- | --- |
| *SMAD2*:NM_001277450.1 | F:5’-TCTGATTCAAAGCAGTGCAGC-3’ R:5’-ACTGGCGTTGGAAGAAGGAAA-3’ | 254 |
| *GSK-3:*NM_032080.1 | F:5’-AGGTGAATCGAGAAGAGCCAT-3’ R:5’-GCCACCAGTTCTCCTGAGTC-3’ | 270 |
| *METTl3*:NM_171992.4 | F:5’-TGTCATGCTGGGCCTTCATT-3’ R:5’-GCGGCTGGCTTTACATTAGC-3’ | 233 |
| *CASP3*:NM_012922.2 | F: 5’-ACCGCACCCGGTTACTATTC-3’  R: 5’-CAAATTCCGTGGCCACCTTC-3’ | 148 |
| *RAGE*:NM_053336.2 | F:5’-GGGTCACAGAAACCGGTGAT-3’ R:5’-GTCTGGGTTGTCGTTTTCGC-3’ | 153 |
| [*HMGN4*](http://www.ensembl.org/Homo_sapiens/Gene/Summary?g=ENSG00000182952.4):XM_033219548.1 | F:5’-ACGCAGATAGAACTGCTTGCT-3’ R:5’-CCGAATTCAAGCCGGAGAGA-3’ | 298 |
| P*TGS1:*NM_017043.4 | F:5’-CGGTACTGCTCACAGATGCT-3’ R:5’-CCGTGCGAGTACAGTCACAT-3’ | 126 |
| *JAK2*:NM_031514.1 | F:5’-GGGTGCCCAGACGAGATTTA-3’ R:5’-AATCCACAACGCTTCCAAAGTC-3’ | 282 |
| [*EIF4B*](http://www.cusabio.cn/target/EIF4B.html):NM_001008324.1 | F:5’-GATGAGGGAGACGACTGCAC-3’ R:5’-GCATTTCTGCTGAATGGAGCC-3’ | 325 |
| *DNMT1B*:NC_037647.1 | F:5’-GCGAGCATGTCAACTAAGCG-3’ R:5’-ATACGGTGCTGATTCCGTCC-3’ | 163 |
| *SIRT1*:NM_001372090.1 | F:5’-TCAGCTGTTGGCTGACTTCAT-3’  R:5’-TCCCAATGCGATGCTGACTT-3’ | 348 |
| *PDCD4*:NM_022265.2 | F:5’-AAGCAAAAAGACGACTGCGG-3’ R:5’-CCACTCTTCATCTCCCCAAGG-3’ | 424 |
| *FOXO1*:NM_001191846.2 | F:5’-GAGCAGTCCAAAGATGCCCT-3’ R:5’-CAGAGCACAGGCAGTACACA-3’ | 395 |
| *ADAM22:*XM_017592951.1 | F:5’-TCTCTTAGGGTCCCAGCTTCT-3’ R:5’-CCGCTTTAGTCTCGGCTTCA-3’ | 218 |
| *KIF5A:*NM_212523.1 | F:5’-GGACACCAAAAGCGCACCT-3’ R:5’-ATAATGACGCTGTCGTCCCC-3’ | 289 |
| *AQP5*:NM_012779.1 | F:5’-CTGGCGGCCATCCTCTATTT-3’ R:5’-CCCCAGCTGAGAGGATGTTG-3’ | 253 |
| *Egr-1*:NM_012551.2 | F:5’-ATCAAAGCCTTCGCCACTCA-3’ R:5’-GTGTAAGCTCATCCGAGCGA-3’ | 196 |
| *HSP27*:M86389.1 | F:5’-CTCGGTTTCCCGATGAGTGG-3’ R:5’-CCACGCCTTCCTTGGTCTTA-3’ | 255 |
| [*MFN2*](http://www.ensembl.org/Homo_sapiens/Gene/Summary?g=ENSG00000116688.12):NM_130894.4 | F:5’-AGCACCCACTGTCTTGTACC-3’ R:5’-GCAAGGTGAGCCTTACAGGT-3’ | 697 |
| *SAMD4A*:XM_017599656.1 | F:5’-AGGCCTAAGTTCCAGTTGCC-3’ R:5’-CTGGAAGTGAGTGTGCCTGT-3’ | 237 |
| [*SEPN1*](http://www.ensembl.org/Homo_sapiens/Gene/Summary?g=ENSG00000162430.12)*:*XM_008764190.2 | F:5’-ATTTCAGACCGGAAAGGGTCC-3’ R:5’-TAGGGGATGGCCAGCATAGA-3’ | 398 |
| *PTEN:*NM_031606.1 | F:5’-CTCAGCCATTGCCTGTGTGT-3’  R:5’-TCAGGGTGAGCACAAGATACT-3’ | 231 |
| *GAPDH*:NM_017008.4 | F:5’-CAGCCCCAGAGTGTGTATCC-3’  R:5’-GAAGATGCGGTCACCTCACA-3’ | 142 |

F: forward primer. R: reverse primer.

|  |
| --- |

**Table S2 Primary antibody information.**

| **Product No.** | **Name** | **MW(KDa)** | **Source/subtype** | **Dilution degree** | **Production company** |
| --- | --- | --- | --- | --- | --- |
| 42544S | RAGE | 52 | Rabbit mAb | 1:200 | Cell Signaling Technology, Inc. |
| 5174S | GAPDH | 37 | Rabbit mAb | 1:200 | Cell Signaling Technology, Inc. |
| 3230S | JAK2 | 120 | RabbitmAb | 1:200 | Cell Signaling Technology, Inc. |
| 9662S | CASP3 | 35 | Rabbit mAb | 1:200 | Cell Signaling Technology, Inc. |
| 4154S | EGR-1 | 75 | Rabbit mAb | 1:200 | Cell Signaling Technology, Inc. |
| 5339S | SMAD2 | 60 | Rabbit mAb | 1:200 | Cell Signaling Technology, Inc. |

MW: molecular weight.

**Table S3 Pathways enrichment analysis of differently expressed genes in SHR of PGQYD-treated rats.**

| ID | Description | Gene Ratio | Bg Ratio | Enrich_factor | P value | Q value |
| --- | --- | --- | --- | --- | --- | --- |
| ko04512 | ECM-receptor interaction | 25/511 | 85/8503 | 4.89 | 1.23E-05 | 2.86E-05 |
| ko04612 | Antigen processing and presentation | 24/511 | 105/8503 | 3.8 | 8.92E-06 | 1.04E-05 |
| [ko04151](../../../../E:/%25E6%25A1%258C%25E9%259D%25A2%25E6%2596%2587%25E4%25BB%25B6%25E5%25A4%25B9/%25E8%25BD%25AC%25E5%25BD%2595%25E7%25BB%2584%25E5%25AD%25A6%25E8%25B5%2584%25E6%2596%2599/%25E8%25BD%25AC%25E5%25BD%2595%25E7%25BB%2584%25E5%259B%25BE%25E7%2589%2587/BMK_9_html/Gene/../../BMK_5_DEG_Analysis/BMK_3_T10_T11_T12_vs_T16_T17_T18/BMK_2_Anno_enrichment/BMK_3_KEGG_map/ko04151.html) | PI3K-Akt signaling pathway | 46/511 | 375/8503 | 2.04 | 2.38E-06 | 9.26E-05 |
| [ko04010](../../../../E:/%25E6%25A1%258C%25E9%259D%25A2%25E6%2596%2587%25E4%25BB%25B6%25E5%25A4%25B9/%25E8%25BD%25AC%25E5%25BD%2595%25E7%25BB%2584%25E5%25AD%25A6%25E8%25B5%2584%25E6%2596%2599/%25E8%25BD%25AC%25E5%25BD%2595%25E7%25BB%2584%25E5%259B%25BE%25E7%2589%2587/BMK_9_html/Gene/../../BMK_5_DEG_Analysis/BMK_3_T10_T11_T12_vs_T16_T17_T18/BMK_2_Anno_enrichment/BMK_3_KEGG_map/ko04010.html) | MAPK signaling pathway | 35/511 | 281/8503 | 2.07 | 2.87E-05 | 0.000839335 |
| [ko04514](../../../../E:/%25E6%25A1%258C%25E9%259D%25A2%25E6%2596%2587%25E4%25BB%25B6%25E5%25A4%25B9/%25E8%25BD%25AC%25E5%25BD%2595%25E7%25BB%2584%25E5%25AD%25A6%25E8%25B5%2584%25E6%2596%2599/%25E8%25BD%25AC%25E5%25BD%2595%25E7%25BB%2584%25E5%259B%25BE%25E7%2589%2587/BMK_9_html/Gene/../../BMK_5_DEG_Analysis/BMK_3_T10_T11_T12_vs_T16_T17_T18/BMK_2_Anno_enrichment/BMK_3_KEGG_map/ko04514.html) | Cell adhesion molecules (CAMs) | 29/511 | 180/8503 | 2.68 | 9.08E-07 | 5.30E-05 |
| [ko00190](../../../../E:/%25E6%25A1%258C%25E9%259D%25A2%25E6%2596%2587%25E4%25BB%25B6%25E5%25A4%25B9/%25E8%25BD%25AC%25E5%25BD%2595%25E7%25BB%2584%25E5%25AD%25A6%25E8%25B5%2584%25E6%2596%2599/%25E8%25BD%25AC%25E5%25BD%2595%25E7%25BB%2584%25E5%259B%25BE%25E7%2589%2587/BMK_9_html/Gene/../../BMK_5_DEG_Analysis/BMK_3_T04_T05_T06_vs_T07_T08_T09/BMK_2_Anno_enrichment/BMK_3_KEGG_map/ko00190.html) | Oxidative phosphorylation | 6/71 | 169/8503 | 4.25 | 0.002778328 | 0.032077901 |
| [ko04915](../../../../E:/%25E6%25A1%258C%25E9%259D%25A2%25E6%2596%2587%25E4%25BB%25B6%25E5%25A4%25B9/%25E8%25BD%25AC%25E5%25BD%2595%25E7%25BB%2584%25E5%25AD%25A6%25E8%25B5%2584%25E6%2596%2599/%25E8%25BD%25AC%25E5%25BD%2595%25E7%25BB%2584%25E5%259B%25BE%25E7%2589%2587/BMK_9_html/Gene/../../BMK_5_DEG_Analysis/BMK_3_T04_T05_T06_vs_T07_T08_T09/BMK_2_Anno_enrichment/BMK_3_KEGG_map/ko04915.html) | Estrogen signaling pathway | 5/71 | 112/8503 | 5.35 | 0.00236519 | 0.032077901 |
| [ko04213](../../../../E:/%25E6%25A1%258C%25E9%259D%25A2%25E6%2596%2587%25E4%25BB%25B6%25E5%25A4%25B9/%25E8%25BD%25AC%25E5%25BD%2595%25E7%25BB%2584%25E5%25AD%25A6%25E8%25B5%2584%25E6%2596%2599/%25E8%25BD%25AC%25E5%25BD%2595%25E7%25BB%2584%25E5%259B%25BE%25E7%2589%2587/BMK_9_html/Gene/../../BMK_5_DEG_Analysis/BMK_3_T04_T05_T06_vs_T07_T08_T09/BMK_2_Anno_enrichment/BMK_3_KEGG_map/ko04213.html) | Longevity regulating pathway - multiple species | 4/71 | 73/8503 | 6.56 | 0.003148434 | 0.032077901 |
| [ko04713](../../../../E:/%25E6%25A1%258C%25E9%259D%25A2%25E6%2596%2587%25E4%25BB%25B6%25E5%25A4%25B9/%25E8%25BD%25AC%25E5%25BD%2595%25E7%25BB%2584%25E5%25AD%25A6%25E8%25B5%2584%25E6%2596%2599/%25E8%25BD%25AC%25E5%25BD%2595%25E7%25BB%2584%25E5%259B%25BE%25E7%2589%2587/BMK_9_html/Gene/../../BMK_5_DEG_Analysis/BMK_3_T13_T14_T15_vs_T16_T17_T18/BMK_2_Anno_enrichment/BMK_3_KEGG_map/ko04713.html) | Circadian entrainment | 13/304 | 103/8503 | 3.53 | 7.14E-05 | 0.007407254 |
| [ko04940](../../../../E:/%25E6%25A1%258C%25E9%259D%25A2%25E6%2596%2587%25E4%25BB%25B6%25E5%25A4%25B9/%25E8%25BD%25AC%25E5%25BD%2595%25E7%25BB%2584%25E5%25AD%25A6%25E8%25B5%2584%25E6%2596%2599/%25E8%25BD%25AC%25E5%25BD%2595%25E7%25BB%2584%25E5%259B%25BE%25E7%2589%2587/BMK_9_html/Gene/../../BMK_5_DEG_Analysis/BMK_3_T10_T11_T12_vs_T16_T17_T18/BMK_2_Anno_enrichment/BMK_3_KEGG_map/ko04940.html) | AGE-RAGE signaling pathway | 56/511 | 477/8503 | 7.46 | 1.02E-08 | 3.41E-07 |

ID: KEGG pathway ID; Description: KEGG pathway name; generatio: gene proportion of the pathway in the gene concentration of interest; Bg Ratio: gene proportion of the pathway in the background gene concentration; enrich factor: enrichment factor; P value: enrichment significance p value; Q value: enrichment significance Q value.
